# Tactic Behaviors in Asymmetric Pacman Particles

**DOI:** 10.1002/smll.75865

**Published:** 2026-09-24

**Authors:** Daniel Gordon, Jeremie Marc Bertrand, Zuyao Xiao, Amir Sheikhshoaei, Sankha Shuvra Das, Gilad Yossifon, Sara Bonella, Ignacio Pagonabarraga, Juliane Simmchen

**Affiliations:** ^1^ Pure and Applied Chemistry University of Strathclyde Glasgow UK; ^2^ Centre Européen de Calcul Atomique et Moléculaire (CECAM) Ecole Polytechnique Fédérale de Lausanne Lausanne Switzerland; ^3^ Freigeist group Physical Chemistry Technische Universität Dresden Dresden Germany; ^4^ Schools of Mechanical & Biomedical Engineering Tel Aviv University Tel Aviv‐Yafo Israel; ^5^ Universitat de Barcelona Institute of Complex Systems (UBICS) Universitat de Barcelona Barcelona Spain; ^6^ Department of Condensed Matter Physics Universitat de Barcelona Barcelona Spain

**Keywords:** active matter, chemotaxis, morphology, phototaxis, simulation

## Abstract

Self‐asymmetric pacman shaped ${\text{TiO}}_{2}$ colloids generate motion through degradation of $H_{2}O_{2}$. This process is modeled using Computational Fluid Dynamics (CFD) to characterize the response and attempt to understand the swimming orientation. In simulation, using μ; the phoretic mobility of the particle, κ; the production rate of the particle and α, the initial angle of the particle, these Pacman particles orientate their bodies according to self‐generated fluid flows. Furthermore, the ability of Pacman particles to respond to chemical and light stimuli is studied in experiments. In particular, these particles are able to sense and reorientate their bodies in chemical and light gradients, allowing their motility to guide them in specific directions.

## Graphical abstract

The swimming behavior of TiO_2_ Pacman particles exhibits different modes depending on the fuel and illumination conditions. However, the behavior of these intrinsically asymmetric particles is still not well understood, and here we present a first approach to modeling their dynamics. Furthermore, we investigate their fundamental responses to some of the most common external stimuli, including chemical gradients, light, and flow.

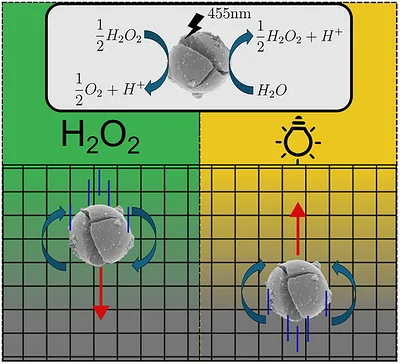

## 1 Introduction

Active matter translates energy from its surroundings into directional motion and can occur over a broad range of length scales. At the smallest scales active matter is constituted by bacteria, algae, and other tiny moving particles, including artificial active particles. Due to its ease of modeling and its reliable swimming properties, one of the most explored experimental cases of an active particle is the catalytic Janus particle. In its most common form, first presented in 2007, a Janus particle is composed of two halves, an inert side and a catalytic Pt side that can break down $H_{2}O_{2}$ fuel into product molecules [1]. While the basic concept had been suggested, prior to experimental realization [2] the theory started to explore the details of this particles swimming mechanisms: the asymmetric distribution of product materials around the particle creates a phoretic force that moves these product molecules to regions of low concentration, this includes a slip velocity that carries the material. In reaction to this, the particle moves in a specific direction determined by the characteristics of the surface material, solvent and product molecules. Charges and ions [3] can also play a part in determining the colloids interaction with its fuel and resulting product molecules. Theory and simulation work has established a framework for such systems. To include all required parameters that influence the complex behaviors, a Boundary Element Method (BEM) was established more than a decade ago [4, 5]. Over time, predictions were refined by tuning parameters in sync with experiments such as wall interactions [6]. The correct prediction of these wall interactions led to a refinement of the b‐values in the BEM model. Further repeated interactions with experiments enabled the improvement of active matter theories and improved our understanding of these systems, for example using flow [7], then adapting theories further when new experimental data became available, specifically on microfluidics [8]. This led to a fairly versatile system able to capture a variety of behaviors.

In the meantime, experimentalists have broadened the range of available materials–not only in terms of shape and size [9, 10], but also in terms of their swimming mechanisms. One example of an experimental swimmer exploiting unique morphologies are Pacman particles [11, 12]. Pacman particles are dehisced, mesoporous ${\text{TiO}}_{2}$ particles which swim in a fuel source, usually $H_{2}O_{2}$. ${\text{TiO}}_{2}$ is a photocatalytic material that can degrade $H_{2}O_{2}$ fuel into oxygen and water when illuminated with UV light. The approach to fabrication [11, 13] removes several bottlenecks in the fabrication process compared to the Janus fabrication: being a one pot synthesis that does not require sputtering of noble metals allows the Pacman particles to be produced at large scale in bulk. In considering scalability, the design of the Pacman particle is unique against other common microswimmers like the Janus particle. The Pacman particles have been used previously in the field of active matter, as they show translational motion when exposed to UV light in a fuel of hydrogen peroxide. They do not require two distinct hemispheres of material to generate motion, the asymmetry necessary to move product molecules stems from the crack across the particles' surface. This leaves the Pacman particles well outside the norm of current modeling approaches, meaning new challenges in simulating their behavior. The exact mechanism responsible for the Pacman particles motility is unknown at this time. Previous studies have demonstrated the difficulty in determining the phoretic mechanism involved in active particle motility [14] and found that multiple mechanisms often play a role (diffuso‐ and electro‐phoresis) with one mechanism dominating. We hypothesise that Pacman particles likely employ a combination of mechanisms, similar to Au@TiO_2_ Janus particle, with whom they share the primary material component. In future works we hope to narrow down this determination and attempts at excluding confounding mechanisms can be found in Supporting Information. In this work, particularly in our simple models, diffusophoresis is assumed to be the dominant mechanism. In addition to their motile capabilities these Pacman particles have been shown to degrade microplastics through the generation of radicals during the redox reaction, creating a destructive environment for microplastic particles. This effect couples with the previously mentioned raft generation and allows Pacman particles to collect and degrade microplastics in the form of micrometer polystyrene beads up to 28% [12].

Here, we aim to combine experimental studies on the swimming properties of Pacman particles with a tailored theoretical understanding. A suitable method must handle complex fluid flows around the particle as well as the influence of those flows on a large solid structure featuring irregular geometries. For this mesoscale problem, the Lattice Boltzmann Method (LBM) is best suited [15, 16]. Derived from the lattice gas automata method this algorithm provides excellent conditions for flows near walls using a bounce‐back scheme for nodes near boundaries that reflects incoming fluid and provides the necessary no‐slip boundary condition [17]. This is essential for effectively computing flows around the Pacman particles body, especially the crack. Furthermore, the LB method is highly parallelisable, making it an excellent candidate for these complex fluid dynamics simulations. In order to refine our understanding of the correct parameters to use in our simulations, we expand our experimental studies beyond pure swimming behaviors and perform further experiments in different conditions with the objective of feeding our results into the simulations to refine our understanding important variables like μ and κ.

## 2 Results

### 2.1 Motion

Previous studies on the Pacman particles have been conducted in an inverted microscope, with viewing and illumination from below the sample stage [11]. In these cases the particles adopt a stable “mouth‐down” swimming position, shown in Figure 1a. In this position the Pacman particles settle at a constant position above the substrate surface (separated by a lubrication layer) and display a range of behaviors that depend on fuel concentration, including the formation of particle rafts [12]. Here the particles swim in long, relatively straight tracks and reorientate randomly (Figure 1b). Particles respond predictably to increasing fuel concentration with increasing velocity (Figure 1c).

**FIGURE 1:**
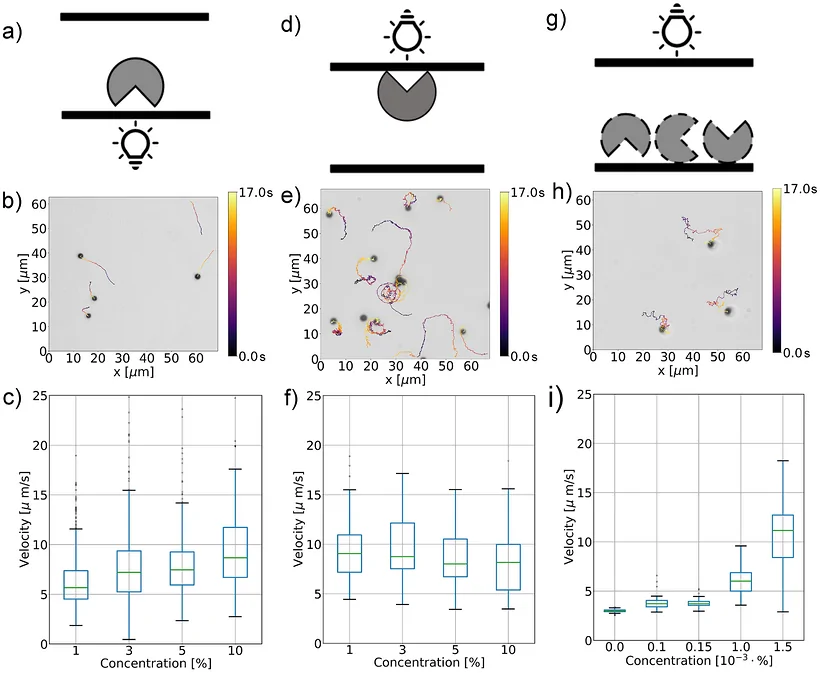
(a–c) Swimming data with bottom illumination. (a) Schematic b) Example particle tracks. (c) Box‐plot illustrating particle fuel concentration dependent velocity. (d–f) Swimming data with top illumination at high fuel concentrations. (d) Schematic displaying the particles' swimming position on the top surface. (e) Particle tracks showing longer, more jagged tracks. (f) Box‐plot illustrating particle fuel concentration dependent velocity showing that velocity remains steady. (g–i) Swimming data with top illumination on the lower substrate at low fuel concentration. (g) Schematic (h) Particle tracks showing similarly longer, more jagged tracks, now with more frequent reorientation. (i) Box‐plot illustrating fuel dependent velocity. At a far lower overall fuel concentration, particle velocity steeply increases with increasing fuel.

However, further experimentation in different microscopic and illumination set‐ups revealed that the Pacman particles' swimming position is heavily dependent on the conditions (see Figure 1d–i). In fact, in an upright microscope with illumination from above, the particles have two distinct swimming positions. At a low concentration of $H_{2}O_{2}$ the Pacman particles are held on the lower substrate by gravity, they do not have enough energy to detach, and therefore swim in the vicinity of the lower substrate. At a sufficiently high concentration of fuel the Pacman particles change orientation and detach from the substrate. They follow the direction of increasing light intensity and swim up toward the source, stabilising their swimming position close to the top substrate (see Figure 1d). Upward propulsion is achieved through a combination of lighting effects, the particle crack turning to the light (see Section 2.2), in addition to overcoming an energy threshold set by gravity and interfacial forces (see Supporting Information). At the upper boundary, the particles move and behave in a similar manner to the behavior shown in Figure 1a: Their mouth is oriented toward the light (in both cases the mouth is aligned with the substrate), the swimming speed is not as strongly correlated with the fuel concentration. A representation of this type of motion and its characteristics is shown in Figure 1d–f. A different picture results from lowering the fuel concentration below the limit where particles can outswim gravity: Particles still move ballistically but do not rise to the top substrate and remain at the bottom. We do not observe a strict alignment with either the light or the bottom substrate. We conjecture that their position is unstable and switching, which makes them prone to more frequent reorientation and follow a more jagged path (see Figure 1h and Figure S6). In these circumstances the particle velocity behaves differently, swimming at a velocity that increases as fuel concentration increases (see Figure 1i) but increases over an order of magnitude up to similar velocities as their high concentration counterparts in other illumination set‐ups. This suggests that, although the concentration has been lowered, the Pacman particles have adopted a more efficient (and unverified) swimming orientation that allows achievements of these greater speeds without the expected fuel concentration.

### 2.2 Simulations

To rationalise these observations, we focus on the essential elements of the Pacman particle and propose a minimal model in the framework of a hybrid free energy/Lattice–Boltzmann (LB) code Ludwig [18]. The model includes the Pacman‐shaped geometry with photocatalytic properties, the diffusion of a solute in the fluid, and the vertically‐confined geometry associated with sedimentation. Chemophoresis is accounted for through a slip velocity profile on the surface of the colloid [19]. The Pacman colloid is represented as a set of solid lattice points (or “nodes”) on the LB grid (see Figure 2a and Section S6 and Figure S5) that geometrically represent a three‐dimensional circular sector: essentially, a sphere with a “cutout” or “dent”. This shape is described in more details in Section S6. The objective of the model is to identify the physical mechanisms underlying the observed dynamics using a minimal coarse‐grained description, rather than to provide a fully quantitative fit to a specific experimental realization. Accordingly, the parameters (μ, α, κ, gravity, etc.) are systematically varied to explore the relevant dynamical regimes and establish the robustness of the observed behavior. In diffusiophoretic active matter, the phenomenology is primarily controlled by the relevant dimensionless groups and by the competition between transport, reaction, and phoretic interactions, rather than by an exact one‐to‐one mapping of all physicochemical parameters. This coarse‐grained strategy is standard in the field [20, 21]. Without needing to fine tune parameters the sign and relative magnitude of the phoretic mobility μ can be found. These findings control the transition between attractive and repulsive regimes, and this behavior remains robust across a broad parameter range.

**FIGURE 2:**
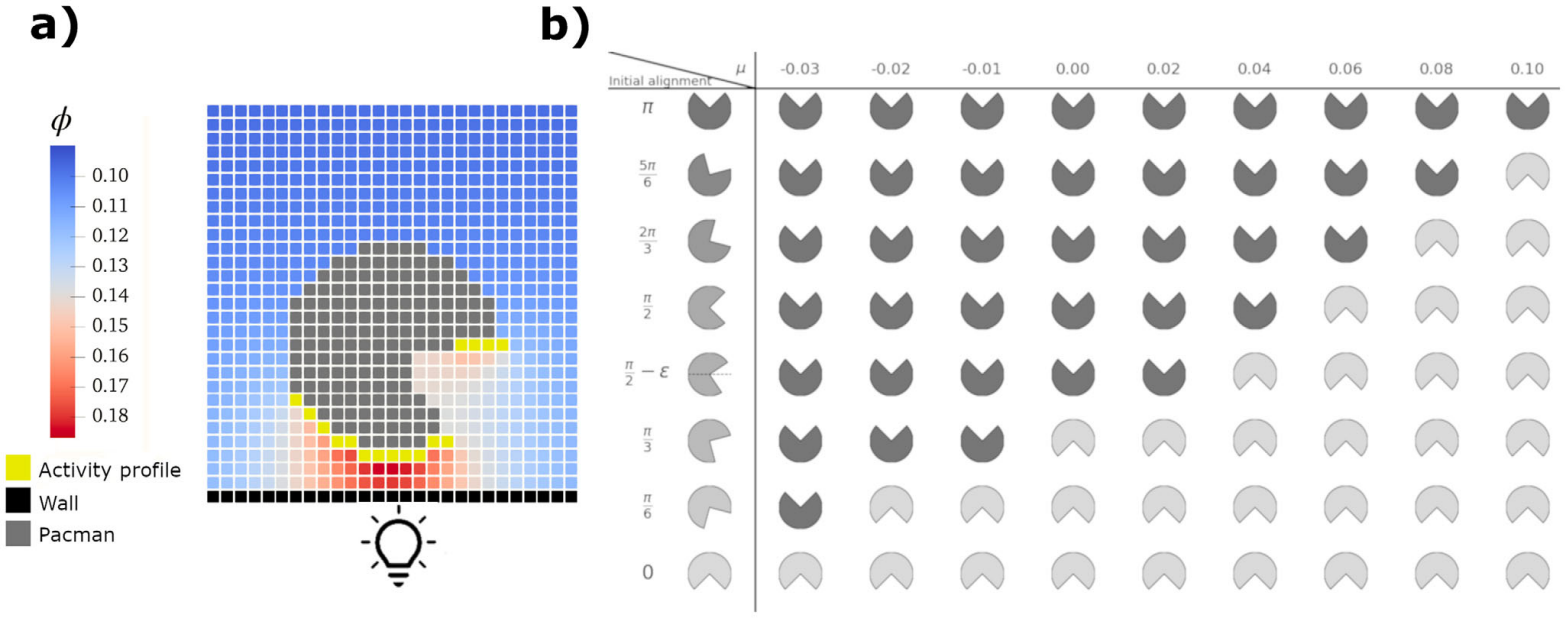
(a) Visual representation of the simulation on a 2‐dimensional slice intersecting the Pacman colloid in its plane of symmetry (see Supporting Information). The solute field ϕ is shown in blue and red, the activity profile in yellow, the solid nodes of the sedimentation wall in black, and those of the Pacman colloid in grey. The illumination light is from below, and the Pacman colloid has an alignment equal to α=π/3. It is initialized with an input radius of $r_{c}=8$ lattice nodes, in the middle of a box of dimensions 60,60,60 nodes. While the upper and lower sides of the box are solid horizontal walls, the sides are connected with periodic boundary conditions mimicking an infinite horizontal space. (b) Final alignment of the colloid as a function of the chemophoretic mobility μ (y‐axis) and the initial alignment (x‐axis). Results indicate that μ<0 favors mouth up (away from light) while μ>0 favors mouth down (toward the light).

A gravitational force is applied on the colloid to account for sedimentation, causing it to move downward toward the lower boundary of the box. To avoid collision between the substrate and the colloid and ensure a proper resolution of the fluid dynamics on the lattice, we impose a colloid/wall lubrication force [17]. As a result, the colloid hovers about two nodes above the floor, as shown in Figure 2a.

We simplify the photo‐degradation of $H_{2}O_{2}$ and ${\text{TiO}}_{2}$ and keep only the production of a single scalar order parameter ϕ that represents the concentration of an effective oxidation product. Its thermodynamic (equilibrium) properties derive from a free energy functional, F[ϕ], of a simplified coarse‐grained chemical field, ϕ, of the form

(1) $$F\left[\phi \right]=\int dr\;\frac{A}{2}\phi^{2}\left(r\right)$$

where A is a positive constant, and r is the position vector on the lattice. The free energy model and its relation to the dynamic of ϕ on the lattice is described in more details in Section S6.

Surface photocatalysis is modeled including source terms for ϕ over fluid nodes immediately adjacent to an activity profile. The latter is defined as the set of solid nodes illuminated by light, which thus become “active”, accounting for shadow effects (the detection of which nodes are blocked from the light and thus remain inactive), as illustrated in Figure 2a, where the light illuminates upward from under the colloid. Shadow effects are clearly visible: The body of the Pacman particle obstructs light thus preventing the nodes within the “mouth” to be activated. At each time‐step, all fluid nodes around active nodes produce ϕ at a rate κ. Thus, in the simulation, we neglect all differences in activity that may exist on the ${\mathrm{TiO}}_{2}$ surface. The free energy together with the surface activity allow us to write down the dynamical equation of ϕ, which follows a Cahn–Hilliard dynamics,

(2) $$\frac{\partial \phi }{\partial t}+\nabla \cdot \left(\phi u\right)=AD\Delta \phi +1_{S}\left(r\right)\kappa$$

where D is the diffusivity, u the local fluid velocity, and

(3) $$\begin{array}{cc} & 1_{S}\left(r\right)\\ & =\left\{\begin{array}{cc} 1 & \text{if the node is immediately under (or above) the activity profile}\\ 0 & \text{otherwise.} \end{array}\right. \end{array}$$

The coupling between the solute and the fluid is determined by the chemical stress that ϕ generates whenever it is locally displaced from its equilibrium state. The associated local force is derived from the local Gibbs–Duhem equation [22]. Based on Anderson's theory of interfacial transport [23], the colloidal motility is accounted for through an effective slip velocity profile on the surface of the colloid, which depends on the local gradient of concentration of solute ϕ [24]. For more details, see Section S6.

We have performed simulations to explore the mechanisms that control the orientation of an illuminated Pacman and predict its orientation, which can be characterized by the angle α (as described in Section S6.1): when α=0 (α=π), the colloid has its mouth facing down (up). To this end, we have put forward a two‐step procedure. First, we reproduce the geometry of the experimental setup (Figure 1a, with light illuminating from below), and vary the phoretic mobility μ and the initial angle of the sedimented colloid. Second, for each value of the aforementioned parameters, we let the fields relax and the colloid re‐align, and measure its final alignment. As shown in Figure 2b, the colloid re‐aligns to one of two stable states: when μ>0, it orients itself with its mouth toward the light, and when μ<0, with its mouth away from it.

Experimental observations, as displayed in Figure 1a, indicate that a sedimented colloid points its mouth toward the light, consistent with what the simulations predict when the phoretic mobility is positive (μ>0) and the gravitational force is stronger than the repulsive phoretic force, which propels the colloid away from region of large concentration of solute.

The simulation results rule out that the Pacman has a negative phoretic mobility; particles would orient with their mouth away from the light source. Also, for μ>0 but weak gravity, Pacman particles would propel away from the light source, inconsistent with experimental observations. Hence, Pacman's alignment together with the fact that the colloid remains sedimented are only consistent with μ>0 and a gravitational force stronger than the chemophoretic force.

Figure 3 compares the Pacman orientation for μ>0 when light is illuminated from the bottom and top, in the experimentally‐relevant parameter range, showing that for a strong gravitational field, the colloid always aligns with the light direction, in agreement with the experimental observations displayed in Figure 1. At low fuel concentration, the self‐propulsion generated by the catalytic reaction is insufficient to overcome gravitational stabilization, leading to reduced particle velocities. Above a threshold fuel concentration, the active phoretic forcing becomes strong enough to sustain vertical swimming, resulting in velocities comparable to those observed in Modes 1 and 2. The observed steep increase therefore reflects the onset of persistent active motion rather than a distinct propulsion mechanism.

**FIGURE 3:**
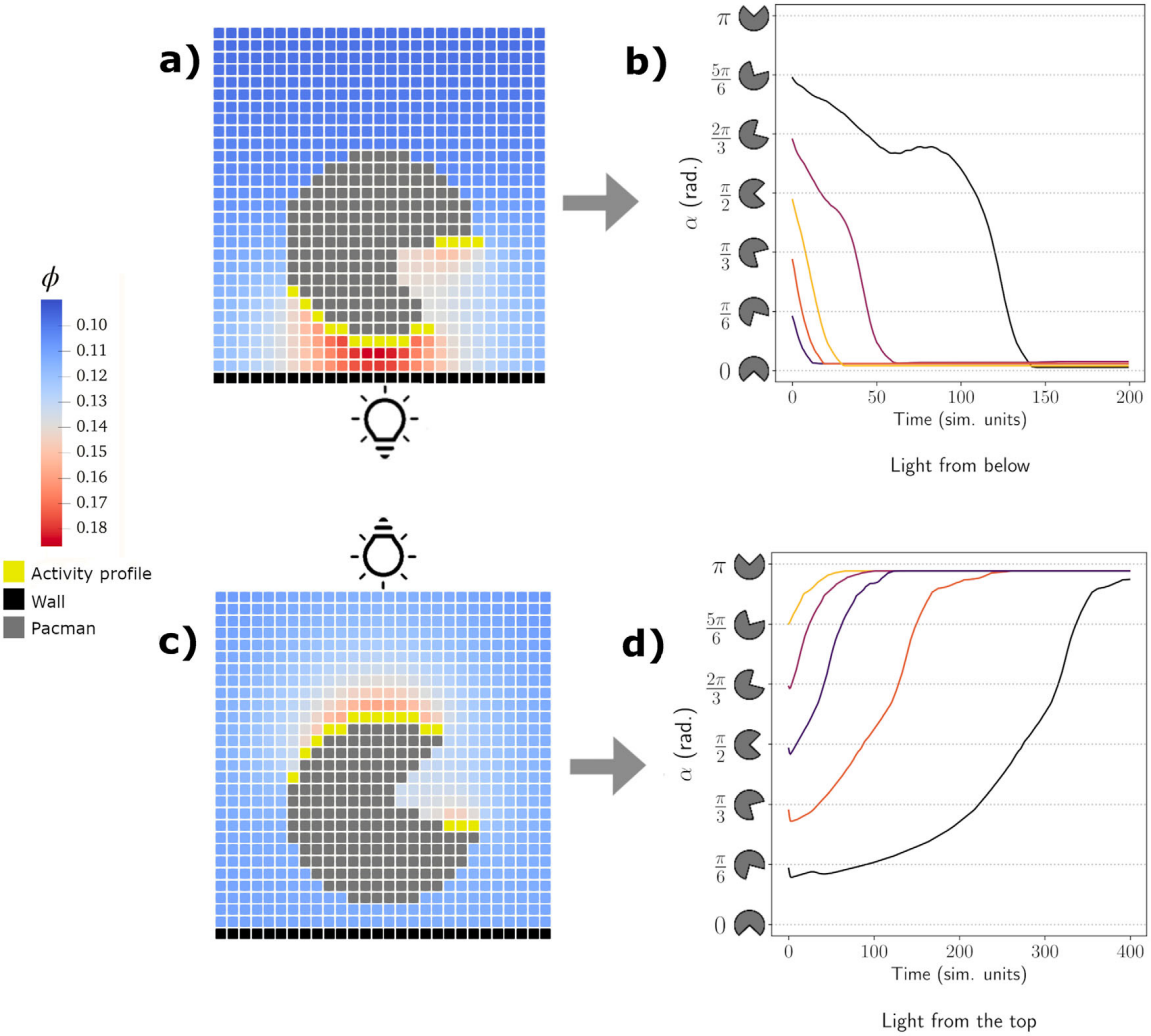
(a) Light illuminates from under, and the colloid is at an angle $\alpha =\frac{\pi }{3}$. (b) Light illuminates from above, and the colloid is at an angle $\alpha =\frac{\pi }{2}$. In each case, the vertical alignment α of the colloid is measured over time, for different initial alignments. The resulting graphs are shown in (b) and (d).

### 2.3 Stimuli Responsive Behaviors

Having gained a solid understanding of the possibilities of Pacman particles behavior and with a model at hand, that is able to grasp the main features of self‐induced gradients and morphology dependent swimming orientation, we aim at understanding the behavior of these dehisced particles in different settings.

#### 2.3.1 Pacman Particles Show Negatively Chemotactic Behavior

To understand the Pacman particles behavior in a chemical fuel gradient they were introduced into a microfluidic chip. Microfluidic experiments were performed in both bottom‐up and top‐down illumination set‐ups, in concentrations ranging from 0.1% $H_{2}O_{2}$ and 1% $H_{2}O_{2}$ for illumination from above. At higher peroxide concentration, controlled experiments in a closed chip were not possible due to the disturbances by oxygen bubbles that both influence swimming behavior and obscure view of the particles.

Figure 4 is divided into two halves with details of two of the experimental cases tested in the microfluidic chip: The left side (Figure 4a–d) shows the Pacman particles behavior in the bottom‐illuminated set‐up at high concentrations of peroxide (1%–10% $H_{2}O_{2}$) and the associated analysis while the right side (Figure 4e–h) shows the behavior under illumination from the top, at low concentrations of peroxide (0.1%–1.5% $H_{2}O_{2}$) with particles moving on the lower substrate. Figure 4i,j shows an example of the microscope view of the experiment and an example tracking output with the addition of a Pacman particle SEM image feature the surface crack which influences these swimming behaviors.

**FIGURE 4:**
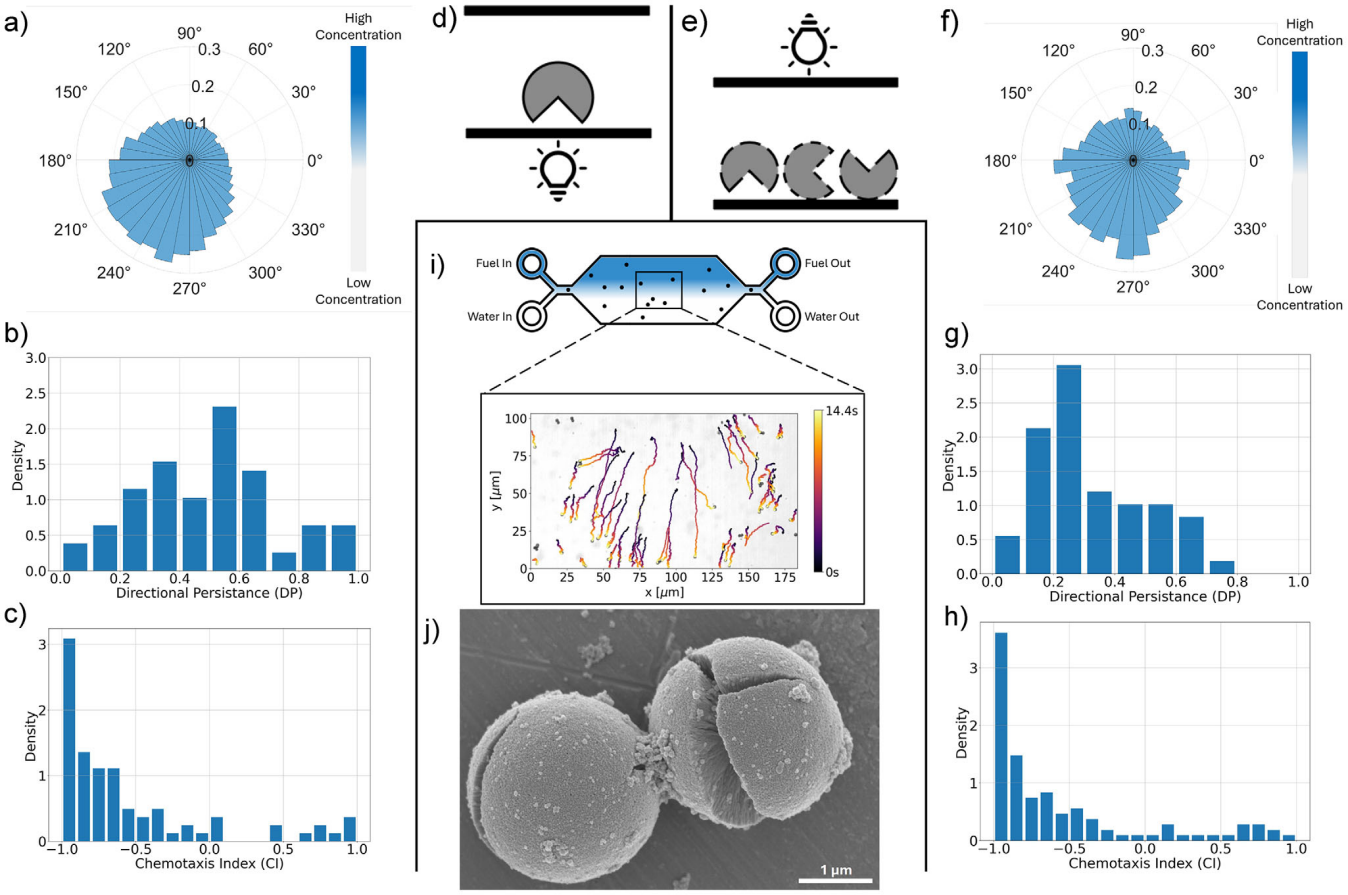
(a–c) Chemotactic swimming behavior in a bottom‐illuminated set‐up. (a) A rose‐plot (polar histogram) showing the preferential swimming direction of the Pacman particles. The number of frames the particles spend swimming at a particle angle is shown. (b) The Directional Persistence (DP) of the Pacman particles, showing a high value (0.6). (c) The Chemotaxis Index (CI) of the particles, showing a strong trend toward –1. This shows that despite the frequent directional changes the Pacmen particles are negatively chemotactic. (d,e) A representation of the two key illumination modes (bottom‐up and top‐down). (f–h) Swimming behavior in a top‐down illuminated set‐up. (f) A rose‐plot of the swimming direction in the top‐down illuminated set‐up showing a strong similarity to the behavior in (a). (g) The DP of the Pacman particles in the top‐down set‐up, showing a value (0.2) suggesting that the particles often reorientate while swimming in this swimming mode. (h) The CI of the particles, showing a similarly strong trend toward –1, implying that despite the more frequent orientation, particles remain negatively chemotactic. (i) A schematic of the stopped‐flow microfluidic channel with an inset showing an example tracking output from the analysis methods. (j) An SEM image showing the Pacman particles, highlighting the crack as a main feature of the particles surface.

To quantify the direction of the swimming, a rose‐plot was generated by quantifying the number of frames traveling at a particular angle using MATLAB (see Figure 4a,f). The height of the bars distributed around the circle show the number of frames a particle spent swimming in a particular direction throughout a recording. In Figure 4, the particles are shown with a preferential motion toward lower $H_{2}O_{2}$ concentration, which suggests the Pacman particles are negatively chemotactic.

In order to further quantify the behavior of the particles and present comparable data for the chemotaxis experiments, dimensionless quantities, the Directional Persistence (DP) and Chemotaxis Index (CI) of the particles are displayed in Figure 4b,c,g,h for each illumination set‐up.

(4) $$DP=\frac{Displacement}{Distance}$$

(5) $$CI=\frac{Displacement_{y}}{Distance}$$

The DP quantifies how long the particles remain moving in a single direction before reorientation. The CI is a similar quantity but takes the ratio of exclusively the Y‐component of the displacement (the axis of the chemical gradient) against the total distance traveled by the particle [25].

The set‐up orientations are shown in Figure 1d,e (for more details please consult Section S2) to demonstrate the particles orientation when tracking and analysis occurs as well as to show the possible orientations the particle could take in the top‐down illumination orientation.

To compare, this analysis shows that when the Pacman particles are illuminated from below they remain chemotactic, as shown by the roseplots and CI for top‐illumination in Figure 4a,c and bottom‐illumination in Figure 4f,h. The CI here is strongly –1 meaning, with the DP, the particles take a negative, jagged path down the chemical gradient. The difference in the swimming position of the particle is displayed in the DP, shown in Figure 4b,g. The bottom‐illuminated set‐up shows the particles remain in a more stable position and so persist in one direction for longer, showing a value of 0.6. Compared to the top‐illuminated orientation, the particles adopts a less stable position which causes more frequent reorientation, resulting in the lower DP value average of 0.2 (See Figure 5).

A no‐light condition was also tested to rule out response of the Pacman particles to simple phoretic effects due to the presence of fuel. This was tested by introducing the particles to an increasing concentration of fuel without UV illumination, removing the active component of the particles interaction with fuel. In this case, no preferential motion was observed (see Section S2).

**FIGURE 5:**
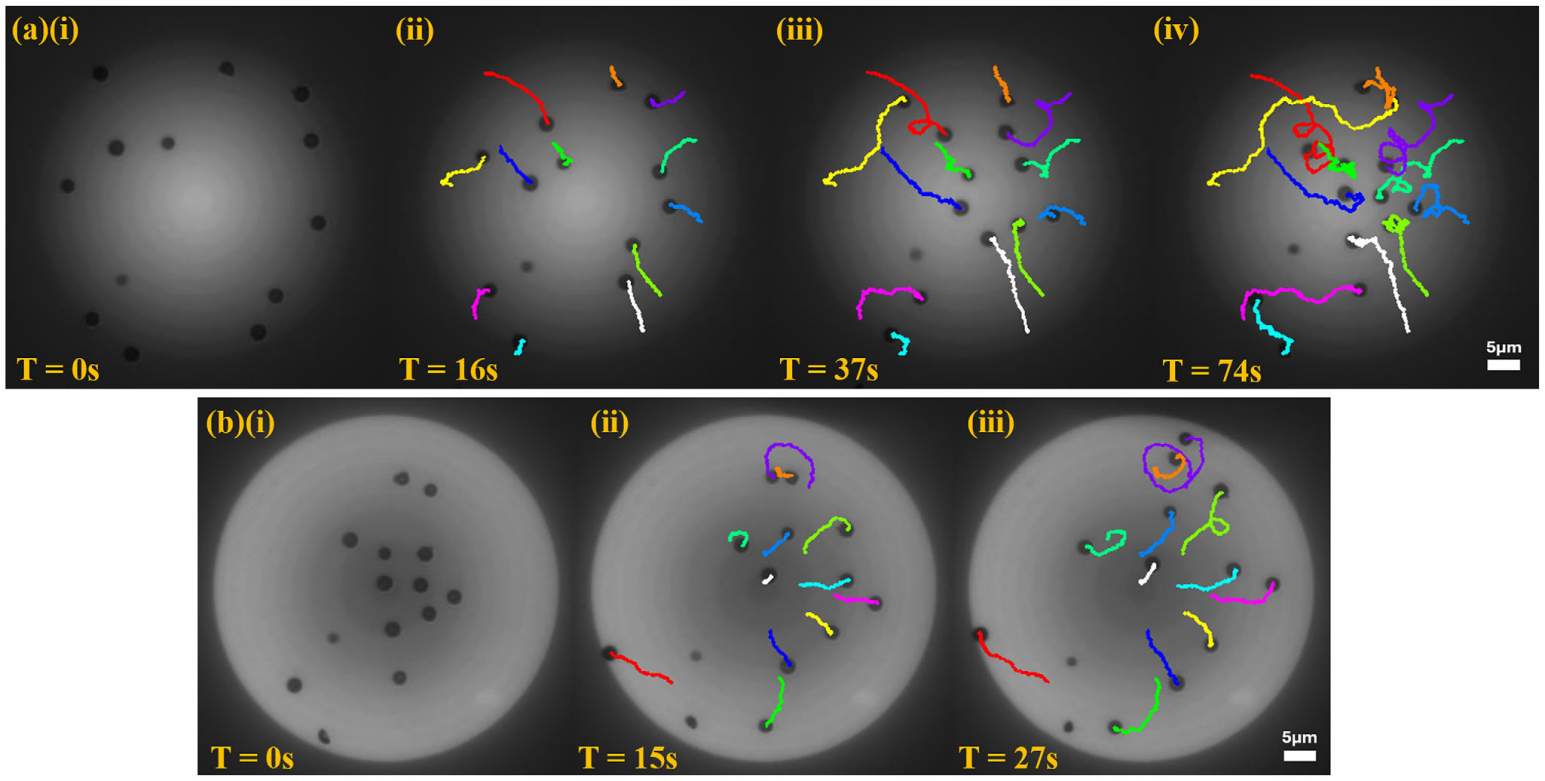
Phototaxis of Pacman micromotor under a radial gradient. (a) Time‐lapsed microscopy images illustrate the motion dynamics of Pacman ${\text{TiO}}_{2}$ micromotors under a radial gradient of UV illumination intensity (a) negative gradient (i–iv), (b) positive gradient (i–iii). The superimposed trajectories highlight their characteristic paths, showcasing directed movement influenced by the light gradient. The Pacman micromotors are dispersed in 5% $H_{2}O_{2}$ solution and illuminated at a maximum light intensity of 1.1$W/{\text{cm}}^{2}$, with the intensity increasing in 10% increments.

#### 2.3.2 Pacman Particles Show Positive Phototactic Behavior

Experiments performed in a microfluidic chamber between two cleaned glass slides showed that Pacman particles in 5% $H_{2}O_{2}$ fuel solutions were strongly influenced by top‐illuminated optical patterns. Both radial and linear illumination gradients (see Movies S3–S5) varied systematically, with the illumination intensity increasing in steps of 10%, from 10% at the minimum to 100% at the maximum, corresponding to maximum light intensities of 1.1$W/{\text{cm}}^{2}$ for the radial pattern (Figure 5, Movies S3 and S5) and 0.79 W/cm² for the linear pattern (Figure S4, Movie S4). This forms a semi‐smooth transitions across the patterns, allowing for the observation of phototactic behaviors of the micromotors in response to spatially varying UV light fields. The Pacman particles showed clear migration patterns toward higher light intensities, independent of the morphology of the gradient. This is very interesting, especially because the behavior of the particles differs between the chemotactic and the phototactic response. While the particles migrate toward lower activity in chemical fields, they propel toward the higher, light induced activities in the light gradients.

#### 2.3.3 Other Responses

Further experimental setups were designed to test the responses of active matter to environmental stimuli [26] including gravity [27, 28], flow [29], surface properties [30] or viscosity [31]. First, flow conditions were tested in the microfluidic set‐up with top‐illumination, exposing the particles to flow speeds of 1–120 μLmin. Particles showed no response to these conditions (see Section S4), indicating that a morphological asymmetry is not sufficient to give a defined response to an external flow.

Experiments on Earth are necessarily influenced by gravity, but testing the gravitational response was performed in a tilted microscope set up (see Section S5) [27, 32]. In this case no response from the particle was observed, likely due to there being no significant difference in the density of the particle over its body.

## 3 Conclusion

In this study we investigate the abilities of a unique microswimmer, the Pacman particle, to swim in different light conditions and respond to external stimuli. Utilizing a non‐standard particle morphology, we show that Pacman particles can generate persistent and directional motion, and we rationalise this behavior With simulations using a Lattice Boltzmann approach. Taking into account the complex shape, we consider the effect of light activation on the Pacman particles, paying particular attention to the effect this particles unique shape plays in influencing its surprising behaviors. Our model evolves the dynamics of a simplified Pacman‐shaped colloid in a viscous fluid, in response to the production and diffusion of a chemical species on its surface and a coarse‐grained representation of interfacial phenomena via the slip‐velocity formulation. We assume a series of parameters in these calculations to reach correct swimming behaviors, which include a μ>0 and a strong gravitational force. Further refinement of these parameters will require additional information about the system. For the case of Janus particles, similar data were obtained in a series of tests in more complex environments [6, 7, 29]. To gather additional information about the Pacman particles in presence of multiple sources of stimulation, we performed experiments in presence of chemical and light gradients, as well as flow and gravity (see Supporting Information for additional stimuli that showed no response from the particles). Interesting behavior is shown for chemical and light gradients that seems to be paradoxically opposed: negative chemotaxis respective to its fuel, i.e., moving to regions of low chemical activity but attracted to high light intensity that increase activity. Taxis performed by the Pacman particles, and additionally this discrepancy in taxis behavior, is not fully explained by the theory that currently covers the popular bimetalic microswimmers [33].

The Pacman particles demonstrate that more complex behavior can be generated without necessarily increasing the complexity of the particles makeup through the inclusion of different materials. The Pacman particles possess the additional advantage of a high throughput synthesis, taking a lead over the comparatively expensive, time consuming and complex synthesis of Janus particles, nevertheless showing a plethora of stimuli responses. This set of observation now enables us to interact with theory, build on existing parameters and refine our knowledge about them to match experimental findings in different environments.

In the future, performing simulations in increasingly more complex environments will allow us to refine our knowledge of these parameters, which will further improve our understanding of the important values for characterizing the behavior of Pacman particles.

## 4 Materials and Methods

### 4.1 Pacman Particles

Pacman particles were prepared by adapting the process described in Lan [13], summarized in detail in Chattopadhyay et al. [11]. Calcination is an important part in it to achieve the crack formation and the crystallinity of the ${\text{TiO}}_{2}$ to give it sufficient activity to actually swim. We have varied different conditions, leading to larger sizes, particles with no crack and different crystal patterns. All particles that showed a crack swim in the same orientation, even if the crack depth varies across different batches, but also within a batch. The factor that led to particles without a crack was actually not the temperature but the concentration of surfactant. However, these particles do not show a nice and consistent swimming behavior due to a lack of asymmetry. A cauliflower particle without a crack but significant asymmetry in the surface shows more random runs. In this report, 2 μm, spherical, rutile ${\text{TiO}}_{2}$ colloids were used. 1.6 g of Pluronic P123 was mixed with 30 mL of tetrahydrofuran, 2 mL acetic acid, and 3 mL of hydrogen chloride. The mixture was stirred until Pluronic P123 was dissolved. Titanium butoxide was then added drop by drop at 18 mL per hour while stirring, followed by 200μL of DI water. The mixture was stirred until it took on a yellow color. The mixture was subsequently heated at 40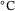 for 24 h, then autoclaved at 70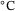 for 24 h. The resulting white powder was washed with methanol and dried at 70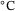 overnight. Finally, the sample was calcinated 350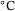 for 3 h under nitrogen atmosphere, then in air at 400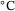 for 3 h. In all experiments the fuel used to move the Pacman particles was hydrogen peroxide, purchased from Fischer Scientific. Originally a concentration of 30%, the $H_{2}O_{2}$ was diluted in DI water to an appropriate concentration for use in the experiments that maintained contact of the Pacman particles to the surface while still creating motion.

### 4.2 Equipment

Videos were taken using a Zeiss Axio Imager.A1. Image capture was performed with a CS2100M‐USB ‐ Quantalux 2.1 MP Monochrome sCMOS camera from Thorlabs. The UV light source was attached to the microscopes in the episcopic position. UV light was supplied from above while white light for viewing was provided diascopically. The camera was coupled to the microscope with a 0.63X Zeiss C‐mount camera adapter. When recording on the Imager. A1 with the Quantalux camera, the ThorCam software was used to visualise and record. When viewing the sample either a LD EC Epiplan–Neofluar 50×/0.55 BD DIC M27 or LD Plan–Neofluar 63×/0.75 Corr Ph2 M27 objective wa s used.

In order to activate the photocatalytic Pacman particles, the particles were illuminated with UV light. Illumination was provided by a UV LED Solo P 365nm led source from Opsytec.

### 4.3 Simulations

The simulation consists of the numerical integration of three different solvers which together solve the dynamical system which represents the complex geometry of the Pacman particle and its coupling with a viscous fluid and a solute. The three parts are (a) the motion of an incompressible viscous fluid (hydrodynamic), (b) a diffusive solute ϕ (thermodynamic) and (c) the colloid. The underlying hydrodynamic is solved with the Lattice–Boltzmann Method with a three‐dimensional D3Q27 set of discrete velocities. The thermodynamic model of ϕ and the motion of the colloid are both solved by finite difference schemes.

### 4.4 Chemotaxis Experiments

These experiments were primarily carried out using equipment purchased from Elveflow, particularly the Elveflow Stop Flow microfluidic set up. This is composed of an OB1 MK3++ pressure controller and a series of 3/2 valves that are controlled by a MUX Wire valve controller. To measure the flow rate two Microfluidic Flow Sensors were used with a range of 2–80 μL/min. A two channel rhombic chamber was used (Rhombic Chamber Chips eP1 ‐ Fluidic 172 microfluidic chip), purchased from Microfluidic Chip Shop, and carries a volume of 120 μL. The chip has a main chamber length of 33.5 mm and a width of 6.5 mm with a total depth of 500 μm. Pressure for the OB1 MK3++ pressure controller was provided by a BOC nitrogen gas cylinder providing 2.5 bar of pressure. Before beginning any expirment, the set‐up uses two channels, one for fuel and one for water plus sample injection. All tubing used was plastic with an inner diameter of 1/32 inches and an outer diameter of 1/16 inches.

Before beginning the experiment, the chip was flushed with water through the sample port to push any old fuel or debris from the chip as best as possible. The illumination in these chemotaxis experiments was a ultraviolet LED pen coupled to the microscope with a wavelength of 365 nm. To control the process the Elveflow Smart Interface (ESI) software was used on a laptop connected to the OB1 MK3++ pressure controller and MUX Wire valve controller. Particles were drawn into a 1 mL syringe compatible with the microfluidic set‐up. Before beginning the flow and pressure cycle described above 0.4 mL of particle solution was pushed into the chamber.

Flow sensors measure flow rate and relay it to the flow controller. Water and fuel is introduced into the chip at a flow rate of 80 μL/min for 30 s to fill the chip to flush any previous solution away, and establish the flow paths that will diffuse together to create a gradient. After this time the valves are switched, squeezing the fluid at 200 mbar to stop the flow and allow material to begin diffusing. When the simultaneous pressure begins the LED is illuminated to begin photo‐catalysis and activate the Pacman particles, measurements are taken at this point. The process is repeated periodically, after 2–4 videos have been taken, to keep the gradient consistent.

### 4.5 Phototaxis Experiments

The microfluidic chamber for phototactic experiments was constructed by sandwiching a spacer layer approximately 120 μm thick (Grace–Bio) between two thoroughly cleaned parallel glass slides. The top glass slide includes two inlet holes, each with a 1 mm diameter, facilitating fluid introduction and removal. For the experiment, Pacman micromotors were dispersed in a 5% $H_{2}O_{2}$ solution. This hydrogen peroxide solution served as the chemical fuel, undergoing catalytic decomposition on the ${\text{TiO}}_{2}$ surface. The UV illumination system utilized a digital micromirror device (DMD)‐based pattern projector (Mightex Polygon 1000‐G) operating at 365 nm LED light source. This system was integrated with an upright microscope (Nikon ECLIPSE Ni‐E) equipped with motorized stages (Marzhauser Scan Plus 100 × 85). Image acquisition was performed using a Prime BSI Express Camera, and a Nikon Fluorescence LED source provided additional illumination flexibility. UV light patterns were projected onto the microfluidic substrate using a 40× Nikon objective lens. The optical power densities of these patterns were measured at the substrate plane with an Ophir Photonics VEGA power meter, ensuring accurate calibration. Motion dynamics of the Pacman ${\text{TiO}}_{2}$ micromotors were characterized under top‐illuminated optical patterns, which included both linear and radial illumination gradients. These gradients were meticulously designed using PolyScan4 software, which allowed fine‐tuned modulation of the UV light intensity. Gradients ranged systematically from 10% at the minimum to 100% at the maximum intensity, forming semi‐smooth transitions across the patterns. The corresponding total intensities ranged from 0.1 $W/{\text{cm}}^{2}$ to 1.1 $W/{\text{cm}}^{2}$ in increments of 0.1 W/cm^2^. This precise control enabled detailed exploration of the phototactic behavior of the micromotors in response to spatially varying UV light fields. To enhance experimental accuracy, the microfluidic chamber design minimized perturbations to the flow and maintained a stagnant flow conditions for motility studies. Additionally, the setup allowed for real‐time observation and recording of micromotor trajectories, providing insights into their propulsion mechanisms under varying photocatalytic conditions.

### 4.6 Analysis

All videos of moving particles were analyzed using python and MATLAB codes to track particles. Particles present in the videos were tracked frame by frame in MATLAB and their paths were represented in rose‐plots showing the number of frames a particle swam at a particular angle. Particle trajectories were tracked in python, also frame by frame, to represent their paths and to calculate velocities.

## Conflicts of Interest

The authors declare no conflicts of interest.

## Supporting information


**Supporting File 1**: smll75865‐sup‐0001‐SuppMat.pdf.

**Supporting File 2**: smll75865‐sup‐0002‐MovieS1.mp4.

**Supporting File 3**: smll75865‐sup‐0003‐MovieS2.mp4.

**Supporting File 4**: smll75865‐sup‐0004‐MovieS3.mp4.

**Supporting File 5**: smll75865‐sup‐0005‐MovieS4.mp4.

**Supporting File 6**: smll75865‐sup‐0006‐MovieS5.mp4.

## Data Availability

The data that supports the findings of this study are available in the supplementary material of this article.
