# Supplementary material for: Tactic Behaviors in Asymmetric Pacman Particles
Source: Small. 2026 Sep 24;22(56):e75865. doi: 10.1002/smll.75865 (PMC13653347; doi:10.1002/smll.75865)
Supplement: Supplementary file 1 [file SMLL-22-e75865-s003.pdf]

Tactic behaviours in asymmetric Pacman particles

*Daniel Gordon, Jeremie Marc Bertrand, Zuyao Xiao, Amir Sheikhshoaei, Sankha Shuvra Das, Gilad Yossifon, Ignacio Pagonabarraga, Juliane Simmchen**

D. Gordon, Dr. J. Simmchen
University of Strathclyde, Pure and Applied Chemistry, 295 Cathedral Street, Glasgow, G1 1XL, UK
Email Address: juliane.simmchen@strath.ac.uk
J. Bertrand, Prof. I. Pagonabarraga
University of Barcelona, Condensed Matter Physics, Gran Via de les Corts Catalanes, 585, L'Eixample, 08007 Barcelona, Spain
Dr. Z. Xiao, Amir Sheikhshoaei
TU Dresden, Freigeist group Phys. Chem., Technische Universität Dresden, Zellescher Weg 19, Dresden, 01069, Germany
S. Shuvra Das, Prof. Gilad Yossifon
Tel Aviv University, School of Mechanical Engineering, Chaim Levanon St 55, Tel Aviv-Yafo, 6997801, Israel

1 List of videos

1. **Movie 1**: An animation of the 3 motions observed when illuminating the Pacman particles from below and above. Graphical representation and analysis is contained the in the main text, Fig. 1.
2. **Movie 2**: An animation of an example video showing the Pacman particles performing chemotaxis and swimming down a chemical gradient of H_2O_2. Graphical representation and analysis is contained in the main text, Fig. 4.
3. **Movie 3, 4, 5**: 3 videos showing the Pacman particles response to differing light gradients; 3 - radial gradient, 4 - linear gradient, 5- alternating radial gradient. Graphical representation and analysis is contained in the main text, Fig. 5 and SI Fig. 4.

2 Details on the chemotactic setup

The setup was presented first in [1]. In summary, it involves two channels that feed into a central chamber, flows for a short distance to allow for the gradient to form and is subsequently stopped precisely using the feedback loop to enable particle observation. Finally, the liquid leaves the chip through two similar output channels. The sample is introduced through the water channel. Illumination was either provided from above using an upright microscope or from below, using an inverted microscope.

3 Behaviour under no illumination

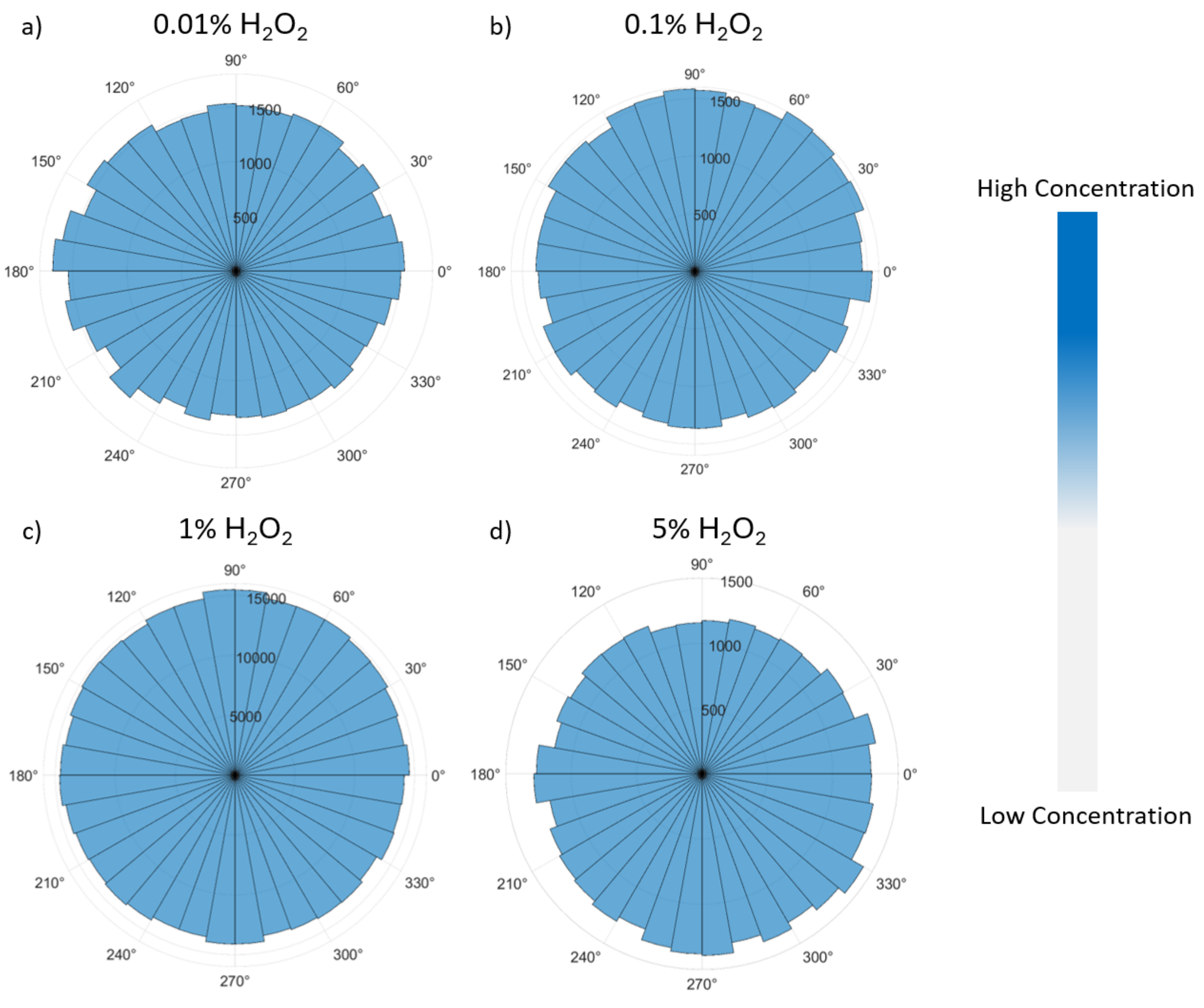

Figure 1: A series of rose plots showing the direction and number of frames particle swim in a) 0.01%, b) 0.1%, c) 1% and d) 5% H_2O_2

The question was then raised whether or not the motion was a form of enhanced phoresis due to the surface properties of the Pacman particle. To test this the particles were introduced into the same microfluidic chip in chemical gradients of varying concentrations of H_2O_2 in a non-illuminated environment, removing the photocatalytic activity. For these experiments no preferential direction was observed in the particles motion as the concentration of fuel increases, as can be seen in SI Figure 1. This implies that the motion of the Pacman particles originates from an interplay been its activity, the presence of the crack and particle orientation.

4 Response to Flow

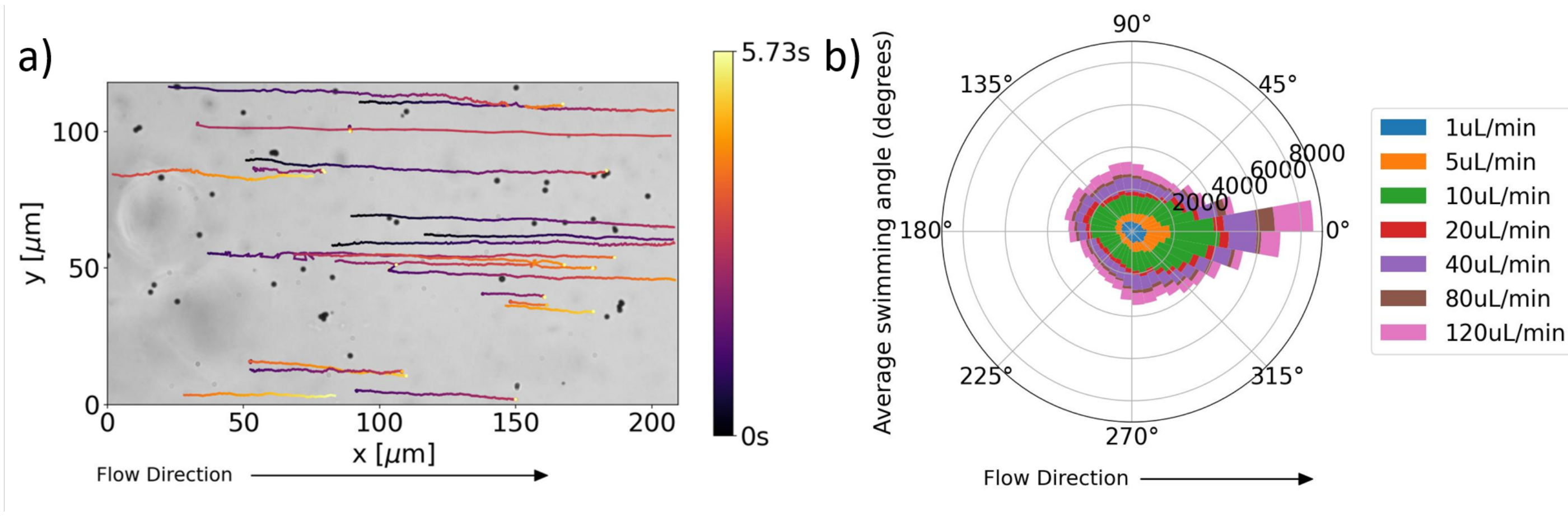

Figure 2: a) An an example of the Pacman particles in 40uL/min flow under UV illumination. The particle are shown flowing in the same direction with the fluid flow. b) A rose-plot showing the average swimming angle in degrees of the Pacman particles. Data was collected at 1, 5, 10, 40, 80 and 120 uL/min and shown together. Consistently all Pacman particles at all flow rates move in the same direction.

5 Motion at an Incline

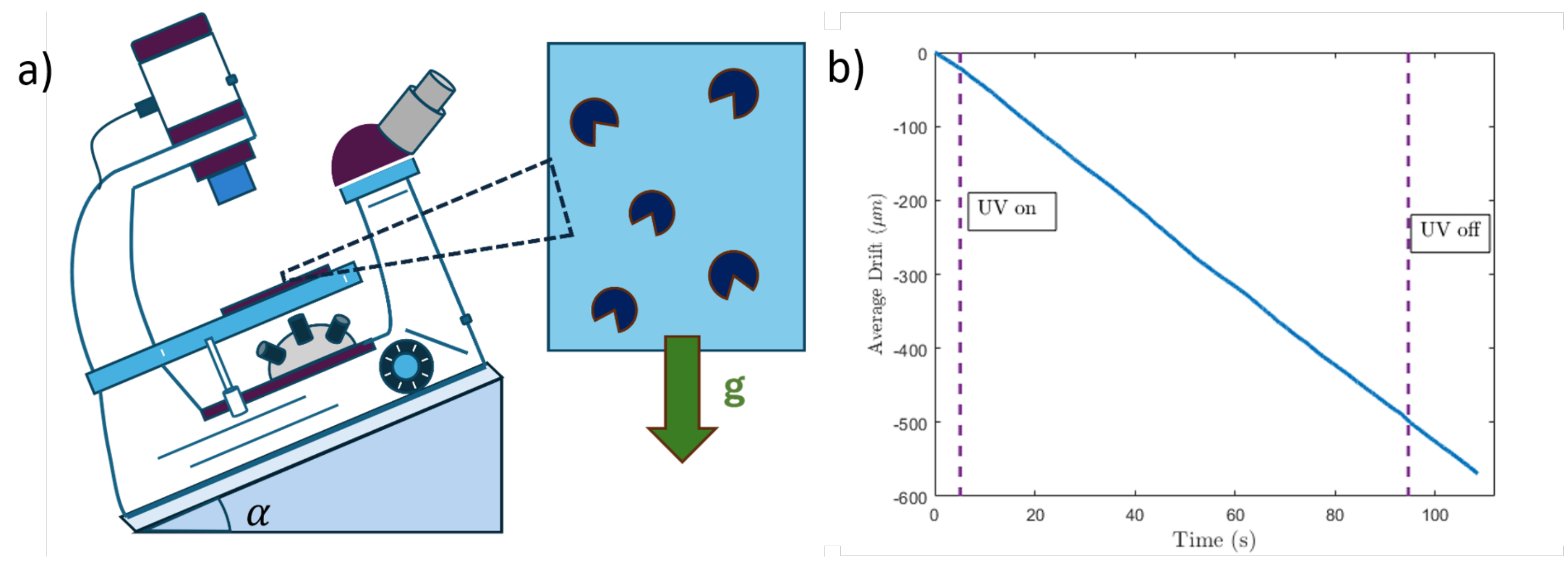

Figure 3: a) A schematic of the gravitaxis setup showing the tilting of the microscope to create a slop to influence particle motion. b) The average drift of the Pacman particles in the setup showing no change in the drift when UV illumination is added.

6 Simulations

6.1 Pacman geometry

The Pacman particle is represented as a set of solid lattice points (or "nodes") on the Lattice-Boltzmann (LB) grid (see Fig. 5a). This shape is reconstructed on the lattice at every step based on the colloid's center position $\mathbf{r_c}$, radius a, orientation vectors $\mathbf{m}$, $\mathbf{n}$, and $\mathbf{p}$, and the mouth opening angle α_m. Throughout this work, we set $\alpha_m = 60°$. A lattice point at position $\mathbf{r}$ is classified as either solid (part of the Pacman particle) or fluid. Specifically, a point is considered solid if it lies within the Pacman-shaped region defined around the center $\mathbf{r_c}$.

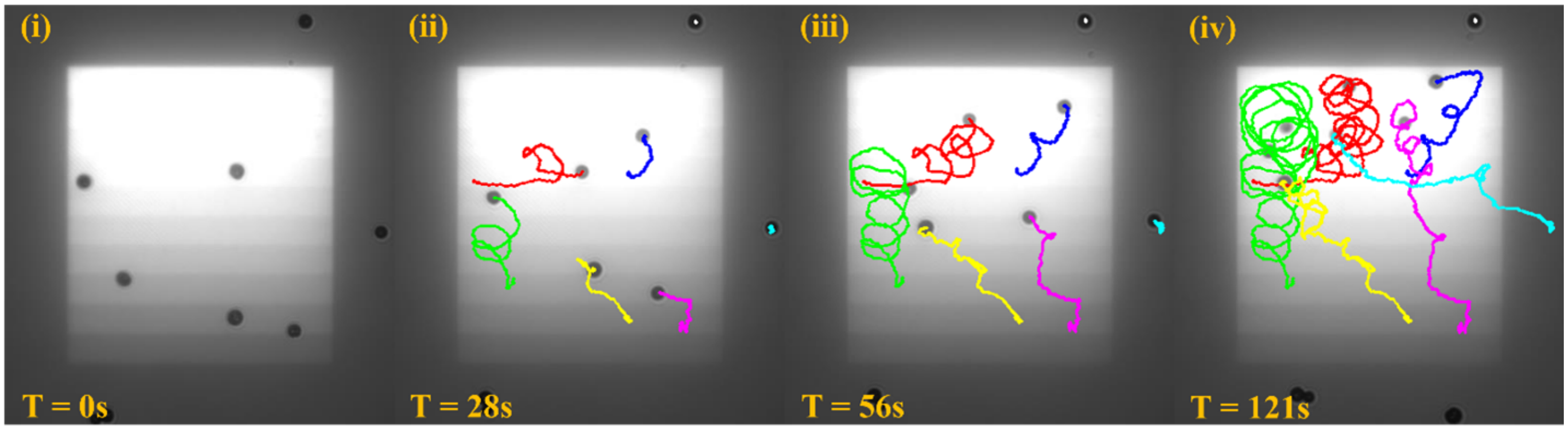

Figure 4: phototaxis of Pac-Man TiO_2 micromotor under a linear gradient. Time-lapsed microscopy images (i-iv) illustrate the motion dynamics of Pac-Man TiO_2 micromotors under a linear gradient of UV illumination intensity. The superimposed trajectories highlight their characteristic paths, showcasing directed movement influenced by the light gradient. The Pac-Man TiO_2 micromotors are dispersed in 5% H_2O_2 solution. Scale bar:??,

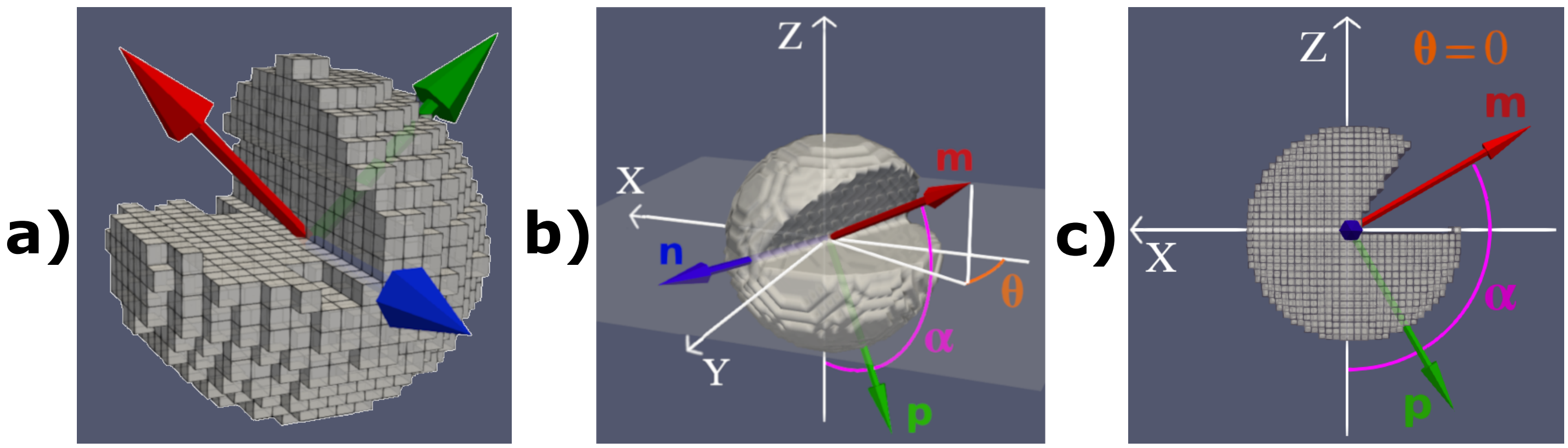

Figure 5: Different visual representations of the Pacman colloid structure. The primary, secondary, and tertiary orientations are shown in red ($\mathbf{m}$), blue ($\mathbf{n}$), and green ($\mathbf{p}$). (a) Solid nodes represented as grey cubes with black outline. (b) A grey iso-surface over the solid node is shown. The orientation vectors are also shown, and an orthonormal cartesian frame (r_c, X, Y, Z) in white, where $\mathbf{r_c}$ is the position vector of the colloid which points to its center. Angles of the spherical frame $(\mathbf{r_c}, \alpha, \theta)$ are shown in pink and orange. (c) Pacman particle in its plane of symmetry $(\mathbf{r_c}, \mathbf{u_x}, \mathbf{u_z})$. The alignment of the colloid is measured by the angle or "vertical alignment" α (in pink).

$$\mathbf{r} \text{ is a solid node} \quad \Longleftrightarrow \quad \begin{cases} (\mathbf{r}-\mathbf{r_c})^2 < a^2 & \text{(inside the spherical boundary)} \\ \arccos\left(\dfrac{(\mathbf{r}-\mathbf{r_c})\cdot\mathbf{m}}{(\mathbf{r}-\mathbf{r_c})\cdot\mathbf{m}+(\mathbf{r}-\mathbf{r_c})\cdot\mathbf{p}}\right) > \dfrac{\alpha_m}{2} & \text{(outside the mouth cutout)} \end{cases} \tag{1}$$

The first condition ensures that $\mathbf{r}$ lies within the spherical boundary defined by the center $\mathbf{r_c}$ and the radius a. The second condition excludes points that fall within the open mouth of the colloid, defined by the opening angle α_m. A visual representation of the solid structure is shown in Fig. 5. The orientation of the colloid is measured by the alignment angle α, shown in Fig. 5c.

6.2 Solute model

All relevant dynamic and thermodynamic quantities can be derived from the free energy functional

$$F[\phi] = \int d\mathbf{r}\, \frac{A}{2}\phi^2(\mathbf{r})$$

In particular, the chemical potetnial is obtained taking the functional derivative with respect to the order parameter

$$\mu = \frac{\delta F}{\delta \phi} = A\phi \tag{2}$$

We consider that ϕ is conserved, and it evolves according to an advection-diffusion equation or Cahn-Hilliard equation [2]:

$$\begin{aligned} \frac{\partial \phi}{\partial t} + \nabla \cdot (\phi \mathbf{u}) &= \nabla \left(D \frac{\delta F}{\delta \phi} \right) \\ &= AD\Delta\phi \end{aligned} \tag{3}$$

where the term on the left is the material derivative of ϕ and the term on the right represents the enthalpic contribution associated with the minimization of F. ϕ has an effective diffusion AD.

6.3 Colloidal phoresis with the slip velocity formulation

Based on Anderson's theory of interfacial transport [3], colloidal motility, or "chemophoresis", is added by imposing an effective slip velocity profile on the surface of the colloid, which depends on the local gradient of concentration of solute $\nabla\phi$.

$$\mathbf{v}_s(\mathbf{r}_s) = \mu(1 - \hat{n} \times \hat{n}) \cdot \nabla\phi \tag{4}$$

where $\mathbf{r}_s$ is a point on the surface of the particle, $\hat{n} = \hat{n}(\mathbf{r}_s)$ is the normal to the surface at $\mathbf{r}_s$, and μ is the phoretic mobility [4].

The prescription of a slip velocity profile on the surface of the colloid results in a violation of the no-slip boundary condition, now replaced with a slip boundary condition. The fluid-solute coupling is further enforced by the bounce-back-on-links procedure, which ensures adequate exchange of momenta between the colloid and the solvent.

7 Variation of angle during swimming

During the motion of the Pacman particles in differing orientation set-ups it is observed that the angle of swimming reorients more frequently. This is visible in the tracks shown in main text, Fig. 1b, e and h. This is also demonstrated in the difference shown in the directional persistence shown in main text, Fig. 4b and g. The Pacman particles, when illuminated from above, take a more jagged path. Here this is further shown in the measure of the Mean-Squared Angular Displacement (MSAD) below. The MSAD shows two distinct regimes around 2 and 3 rad^2 where the top-illuminated conditions exist separately from the bottom-illuminated condition.

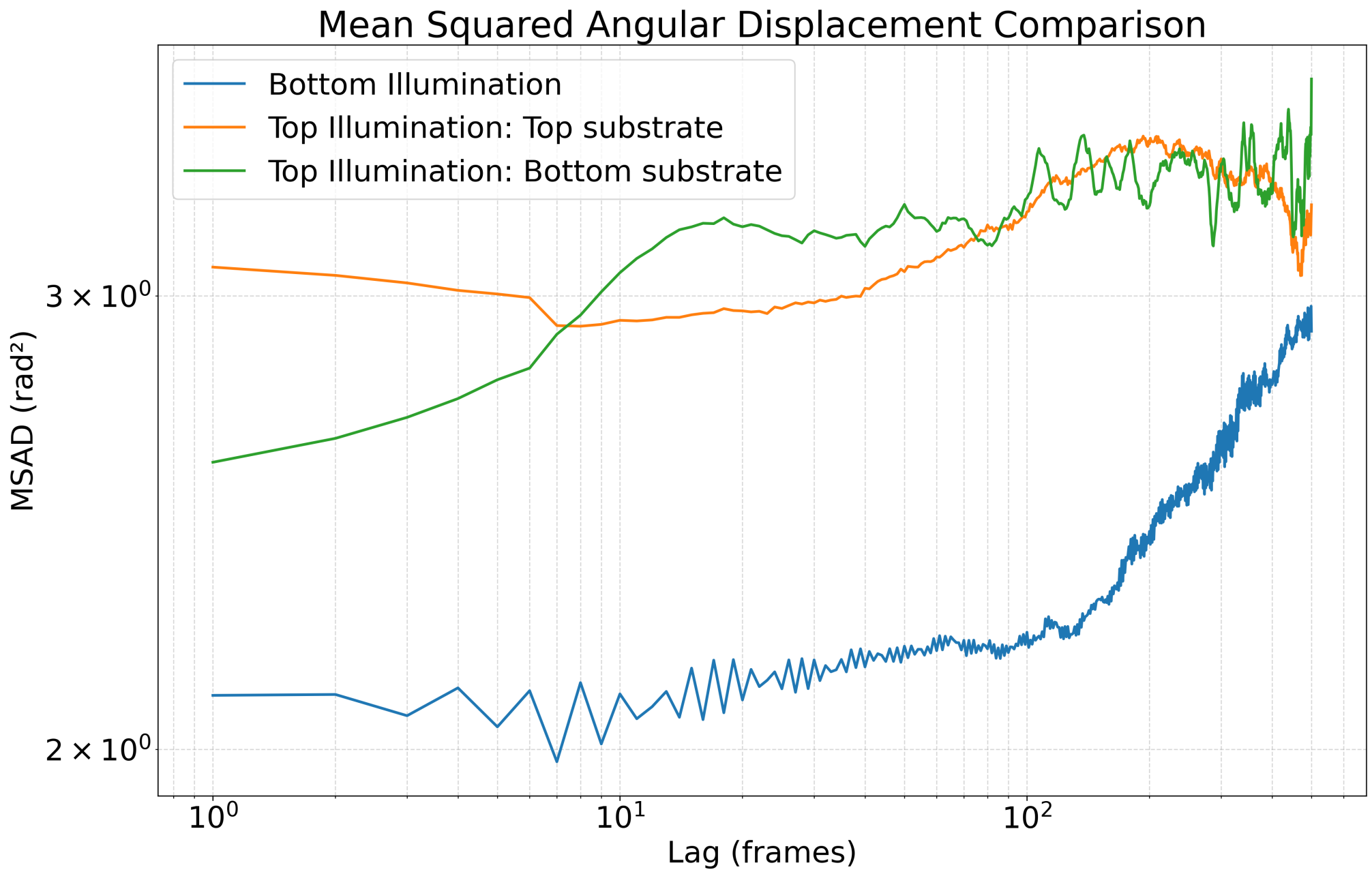

Figure 6: A figure showing the Mean-Sqaured Angular Displacement (MSAD) on a log-log plot. 3 lines show the 3 illumination and swimming conditions demonstrated in main text, Fig.1 (Blue: Bottom illumination, swimming on the lower substrate, Orange: Top illumination, swimming on the upper substrate and Green: Top illumination swimming on the lower substrate). The MSAD shows two regimes, with the top-illuminated conditions existing ~ 3 rad^2 and the bottom-illuminated condition existing ~ 2 rad^2.

References

[1] Z. Xiao, A. Nsamela, B. Garlan, J. Simmchen, *Angewandte Chemie International Edition* **2022**, *61*, 21 e202117768.

[2] S. Thampi, I. Pagonabarraga, R. Adhikari, *Physical Review E* **2011**, *84* 046709.

[3] J. L. Anderson, *Annual Review of Fluid Mechanics* **1989**, *21*, Volume 21, 1989 61.

[4] A. Scagliarini, I. Pagonabarraga, *Soft Matter* **2020**, *16* 8893.
